# DE-PNN: Differential Evolution-Based Feature Optimization with Probabilistic Neural Network for Imbalanced Arrhythmia Classification

**DOI:** 10.3390/s22124450

**Published:** 2022-06-12

**Authors:** Amnah Nasim, Yoon Sang Kim

**Affiliations:** BioComputing Lab., Institute for Bio-Engineering Application Technology, Department of Computer Science and Engineering, KOREATECH, Cheonan 31253, Korea; amnah88@koreatech.ac.kr

**Keywords:** arrhythmia, electrocardiogram, differential evolution, imbalanced class, matthews correlation coefficient

## Abstract

In this research, a heartbeat classification method is presented based on evolutionary feature optimization using differential evolution (DE) and classification using a probabilistic neural network (PNN) to discriminate between normal and arrhythmic heartbeats. The proposed method follows four steps: (1) preprocessing, (2) heartbeat segmentation, (3) DE feature optimization, and (4) PNN classification. In this method, we have employed direct signal amplitude points constituting the heartbeat acquired from the ECG holter device with no secondary feature extraction step usually used in case of hand-crafted, frequency transformation or other features. The heartbeat types include normal, left bundle branch block, right bundle branch block, premature ventricular contraction, atrial premature, ventricular escape, ventricular flutter and paced beat. Using ECG records from the MIT-BIH, heartbeats are identified to start at 250 ms before and end at 450 ms after the respective R-peak positions. In the next step, the DE method is applied to reduce and optimize the direct heartbeat features. Although complex and highly computational ECG heartbeat classification algorithms have been proposed in the literature, they failed to achieve high performance in detecting some minority heartbeat categories, especially for imbalanced datasets. To overcome this challenge, we propose an optimization step for the deep CNN model using a novel classification metric called the Matthews correlation coefficient (MCC). This function focuses on arrhythmia (minority) heartbeat classes by increasing their importance. Maximum MCC is used as a fitness function to identify the optimum combination of features for the uncorrelated and non-uniformly distributed eight beat class samples. The proposed DE-PNN scheme can provide better classification accuracy considering 8 classes with only 36 features optimized from a 253 element feature set implying an 85.77% reduction in direct amplitude features. Our proposed method achieved overall 99.33% accuracy, 94.56% F1, 93.84% sensitivity, and 99.21% specificity.

## 1. Introduction

**Figure 1:**
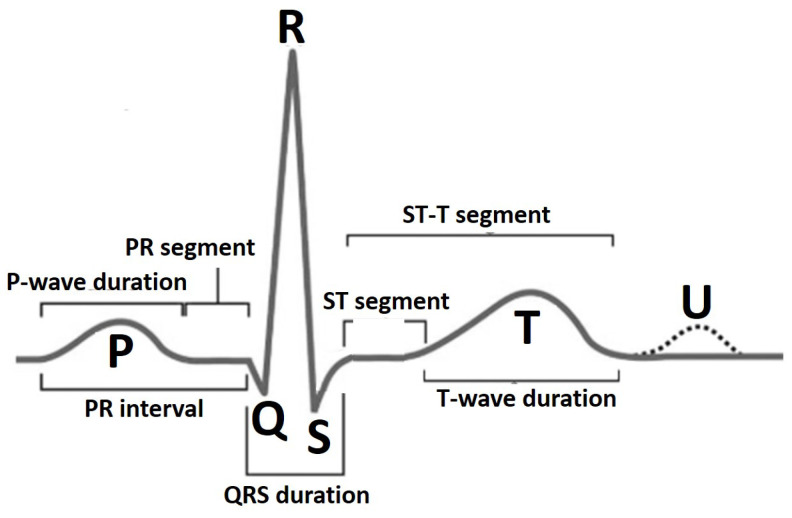
PQRST wave and primary fiducial markers for ECG heartbeat.

An electrocardiogram (ECG) represents electrical activity of the heart in a graphical manner. It is a non-invasive and commonly-used tool by clinicians and cardiology specialists to monitor the function of the heart and diagnose both critical and non-critical heart diseases. The ECG signal is defined by a standard PQRST sequence of waves as shown in Figure 1. The P wave indicates atrial depolarization. The QRS complex consists of a Q wave, R wave and S wave and represents ventricular depolarization. The T wave comes after the QRS complex and indicates ventricular repolarization. Each of these entities, i.e., P wave, QRS complex and T wave (possibly a U wave) have a unique pattern in terms of duration, amplitude and consecutive inter-beat correlation. A deviation in this normal pattern signifies an abnormal event. The diseased state in the case of cardiovascular monitoring is called an arrhythmia. The occurrence of an arrhythmic event is rare but critical and life threatening leading to a sudden cardiac arrest or sudden cardiac death incident. Recently, cardiovascular health monitoring has shifted from traditional in-clinic ECG machines [1,2] to portable and wearable ECG devices [3,4,5] that accumulate 24 h single-lead patient ECG data for long-term and continuous monitoring scenario. Identifying deviating patterns from the normal heartbeats in this large accumulated data is a tiresome and tedious job for clinicians and suffers from inter- and intra-observer variation error. This problem has led to the evolution in the development of computer-aided diagnostic methods for cardiovascular disease pathology indication for early referral to cardiac specialists and initiation of proper and timely medical attention.

Recent developments in the use of wearable ECG devices and on devices based on the Internet of Medical Things (IoMT) have led to an explosion of routinely collected individual ECG data. The use of feature engineering and computational intelligence methods to turn these ever-growing ECG monitoring data into clinical benefits seems as if it should be an obvious path to take. Computer-aided ECG arrhythmia classification systems that use intelligent techniques for the development of smart healthcare monitoring platforms are popular nowadays. A computer-aided early referral arrhythmia classification system [6,7,8] usually involves a feature extraction process in which a set of features is calculated for each individual heartbeat (the type of features used might be hand-crafted, statistical, morphological or spectral, etc.) and classifier construction to learn the features and classify incoming heartbeats. Using all the features calculated in the feature extraction step and a multi-layered classifier not only introduces heavy computational cost but also affects classifier performance due to the presence of redundant/corrupted features. The latest systems deploy a feature reduction/optimization step before classification to remove all unnecessary features. This also allows the use of a single layered or a computationally less intensive learning algorithm for classification.

In the latest competitive research, novel features and various classifiers have been utilized for ECG beat classification tasks. Sayantan et al. [9] feature representation of ECG is learnt using the Gaussian–Bernoulli deep belief network followed by a linear support vector machine (SVM) training in the consecutive phase. Elhaj et al. [10] investigated principal components of discrete wavelet transform coefficients and higher order statistics. Afkhami et al. [11] used parameters of Gaussian mixture modeling together with skewness, kurtosis and 5th moment and applied an ensemble of decision trees to classify the heartbeats using a class-oriented scheme. Liu et al. [12] improved the dictionary learning algorithm for vector quantization of ECG. Shen et al. [13] used wavelet transform-based coefficients, signal amplitude and interval parameters. A new classifier, which integrates k-means clustering, one-against-one SVMs, and a modified majority voting mechanism, is proposed to further improve the recognition rate for extremely similar classes. Qin et al. [14] developed wavelet multi-resolution analysis to extract time-frequency domain features and applied one-versus-one support vector machine to characterize six types of ECG beats. Zhai [15] and Acharaya et al. [16] used a CNN classifier. Oh et al. [17] used CNN and LSTM in combination to propose a refined classification method and generated synthetic data to overcome imbalance problem with accuracies of 94.03% and 93.47% with and without noise removal, respectively.

Recently, researchers have presented different feature reduction methods to reduce the input dimensions of ECG signals for neural classifiers. To name a few of the latest, Zhang et al. [18] extracted statistical features applying a combined method of frequency analysis and Shannon entropy and used information gain criteria to select 10 highly effective features to obtain a good classification on five types of heartbeats. Yildrim et al. [19] implemented a convolutional auto-encoder-based nonlinear compression structure to reduce the feature size of arrhythmic beats. Tuncer et al. [20] applied the neighborhood component analysis feature reduction technique to obtain 64, 128 and 256 features from a 3072 feature vector size. Wang et al. [21] proposed an effective ECG arrhythmia classification scheme consisting of a feature reduction method combining principal component analysis with linear discriminant analysis. Alonso-Atienza et al. [22] used a filter-type feature selection procedure which was proposed to analyze the relevance of the computed parameters. Chen and Yu [23] applied nonlinear correlation-based filters, calculated feature–feature correlation to remove redundant features prior to the feature selection process based on feature–class correlation. Asl et al. [24] proposed the feature reduction scheme based on generalized discriminant analysis. Haseena et al. [25,26] used a fuzzy C-mean (FCM) clustered probabilistic neural network (PNN) for the discrimination of eight types of ECG beats. The performance has been compared with FCM clustered multi layered feed forward network trained with the back propagation algorithm. Important parameters are extracted from each ECG beat and feature reduction has been carried out using FCM clustering. Polato et al. [27] used principal component analysis. Genetic algorithms have also been applied recently for the optimization of ECG heartbeat features [28,29,30,31] and proved to be advantageous in improving the time-cost value in heartbeat classification methods.

Previously proposed automated cardiovascular disease diagnosis systems have mostly followed the design objective of achieving high performance by maximizing accuracy, F1-score, sensitivity and precision measures. A major limitation in the case of general and particularly cardiovascular disease diagnosis is a highly unbalanced ratio or frequency of occurrence of normal to abnormal events. Furthermore, existing multi-class learning approaches mainly focus on exploiting label correlations to facilitate the learning process. However, an intrinsic characteristic of multi-class learning, i.e., class-imbalance [32] has not been well studied [33,34,35]. The Matthews correlation coefficient (*MCC*) was first used by B.W. Matthews for the performance assessment of protein secondary structure prediction [36]. Since then, it has become a widely used performance measure in biomedical research. *MCC* and Area Under ROC Curve (AUC) have been chosen as the elective metric in the US FDA-led initiative MAQC-II that aims to reach a consensus on the best practices for the development and validation of predictive models for personalized medicine [37].

**Figure 2:**
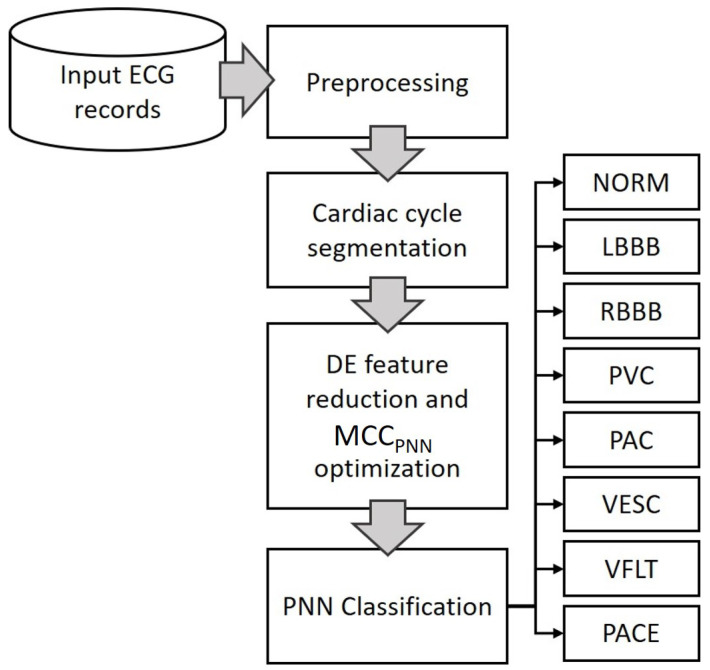
Methodology flowchart.

This research models a metaheuristic search algorithm Differential Evolution (DE) [38] which is a very robust and highly effective heuristic algorithm. Differential evolution has also been used in many applications in many fields. For example, surface and Beizer curve optimization [39], electronic circuitry [40], lithology [41], optimizing solar cells [42] and many others. Although not directly related, these papers should be cited to show the wide range of uses of the differential evolution algorithm. The current work implements DE to optimize direct ECG heartbeat amplitude features to maximize MCC for eight arrhythmia beat classes having imbalanced and uncorrelated class distributions. The algorithm is tuned to find a minimized optimum combination of features that performs better as compared to all features. The motivation here is to remove noisy or redundant signal points, specifically for the task of classification. Classification using PNN is performed with optimum and all features to show the difference. The proposed method is simply depicted in Figure 2. Using PNN for classifying abnormal heartbeats with reduced direct heartbeat amplitude points diminishes the computation of a secondary feature extraction step, produces higher classification performance due to removal of unnecessary features and is faster due to the optimized minimum number of features and less complex PNN learning algorithm. The rest of this paper is organized as follows. In Section 2, the clinical data, cardiac cycle identification and normalization, DE feature reduction and the PNN classification for arrhythmia identification are described in detail. Section 3 includes the performance evaluation measures and data division for training and testing. Results are presented in Section 4. A detailed discussion on the achieved results plus some future possibilities are presented in Section 5.

## 2. Materials and Methods

### 2.1. Clinical Data

**Figure 3:**
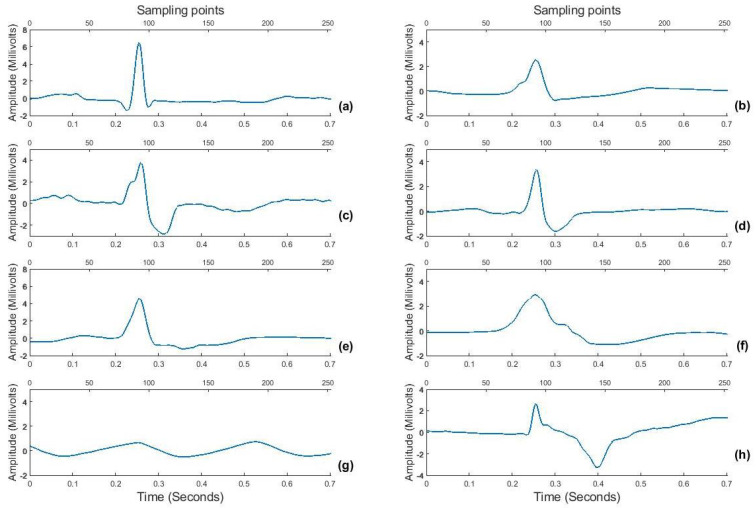
Sample beats for eight ECG beat classes: (**a**) NORM, (**b**) LBBB, (**c**) RBBB, (**d**) PVC, (**e**) PAC, (**f**) VESC, (**g**) VFLT and (**h**) PACE.

ECG data for this study belongs to “MIT−BIH arrhythmia database” developed in 1987 and are available as open source on Physionet (https://physionet.org, accessed on 15 December 2020) [43,44]. The database consists of 48 two-channel ambulatory ECG records, each of approximately 30 min duration digitized at a sampling rate of 360 Hz acquired from 47 subjects out of which 25 subjects were men aged 32 to 89 years, and 22 were women aged 23 to 89 years (2 records came from the same subject). Each record has simultaneous recordings from 2 leads, MLII and V5. For the purpose of testing a wearable ECG sensing scenario that mostly uses a single lead for acquisition [45], this work uses ECG signal from only the MLII lead. Each record is supported by an annotation file providing the R-peak positions and corresponding beat labels (*Lb*). Hence, for this research, 107,800 heartbeats are used having corresponding labels for 8 classes, i.e., normal (NORM), left bundle branch block (LBBB), right bundle branch block (RBBB), premature ventricular contraction (PVC), atrial premature contraction (PAC), ventricular escape (VESC), ventricular flutter wave (VFLT) and paced (PACE) beat. The selected 8 classes include less frequent but clinically significant arrhythmic beats too to prove the validity of the proposed algorithm. An sample of all beat patterns is shown in Figure 3 as an example.

### 2.2. Proposed Methodology

**Figure 4:**
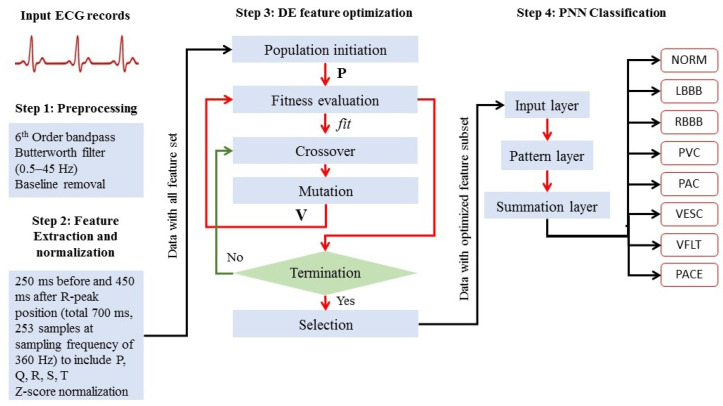
Detailed methodology: Differential Evolution-based feature optimization with Probabilistic Neural Network for imbalanced arrhythmia classification.

The proposed methodology as graphically shown in Figure 2 and in detail in Figure 4 is explained in four steps; (1) preprocessing, (2) cardiac cycle identification and normalization, (3) feature optimization, and (4) disease-based classification as follows:

#### 2.2.1. Preprocessing

In the preprocessing stage, power and low-frequency components are removed from the raw ECG signal by using a 6th-order bidirectional Butterworth band-pass filter with lower and upper cut-off frequencies of 0.5 and 40 Hz, respectively. The baseline is computed as a cubic spline interpolation of fiducial points placed 90 milliseconds before R-peak positions as an approximation for baseline PR-segment and subtracted from the bandpass-filtered signal.

#### 2.2.2. Cardiac Cycle Identification and Normalization

**Figure 5:**
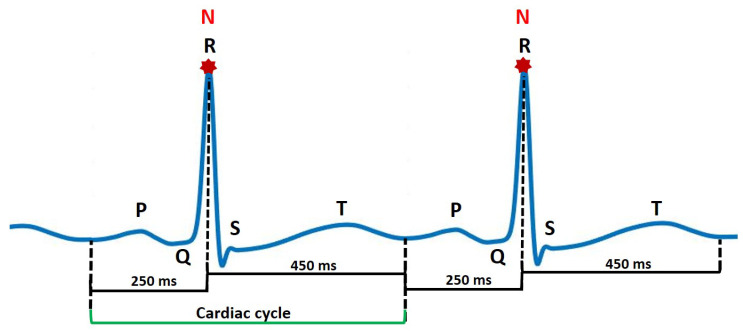
Cardiac cycle identification.

Using the R-peak positions provided with each record, a heartbeat sample is identified as having an onset of 250 ms before each R-peak position to 450 milliseconds after each R-peak position. This definition makes each heartbeat consist of 253 sampling points and ensures that the important characteristic points of ECG such as P, Q, R, S, and T waves are included [46] as shown in Figure 5. The signal amplitude biases in the waveforms of the ECG beat samples are inconsistent due to instrumental and human errors. Hence, we utilize the Z-score method to reduce the above-mentioned differences in each ECG beat sample. Through the Z-score method, the mean value of each ECG sample is first subtracted from each ECG sample to eliminate the offset effect and then divided by its standard deviation [21]. This procedure results in a normalized ECG beat sample with zero mean and unity standard deviation. Figure 3 shows samples for all 8 ECG beat classes used in this research.

#### 2.2.3. Feature Optimization

The mathematical model followed for feature optimization using DE to find the minimum number of features that result in maximum classification performance is explained as follows.

##### Population Initiation

An initial population matrix **P** is generated as in Equation (1) to represent the possible solution/optimization space consisting of $n_{p}$ number of binary row vectors **p** called population individuals each of length $n_{f}$ (number of features in heartbeat samples in this case 253 as mentioned in Section 2.2.2).

(1) $$P_{n_{c},n_{f}}=\left[\begin{array}{c} p_{1}\\ p_{2}\\ .\\ .\\ p_{i}\\ .\\ .\\ p_{n_{p-1}}\\ p_{n_{p}} \end{array}\right]=\left[\begin{array}{cccccc} p_{1,1} & p_{1,2} & . & . & . & p_{1,n_{f}}\\ p_{2,1} & p_{2,2} & . & . & . & p_{2,n_{f}}\\ . & . & . & \; & . & .\\ . & . & . & \; & . & .\\ p_{i,1} & p_{i,2} & . & p_{i,j} & . & p_{i,n_{f}}\\ . & . & . & \; & . & .\\ . & . & \; & . & . & .\\ p_{n_{p-1,1}} & p_{n_{p-1,2}} & . & . & . & p_{n_{p-1},n_{f}}\\ p_{n_{p,1}} & p_{n_{p,2}} & . & . & . & p_{n_{p},n_{f}} \end{array}\right]$$

where, $p_{i,j}$ represents bit value at $j^{th}$ feature position in $i^{th}$ population individual. Here, j=1 to $n_{f}$ and i=1 to $n_{p}$. 1’s and 0’s in each population individual represent the selected and non-selected features, respectively. $p_{i,j}$ for $p_{1}$ to $p_{n_{p-1}}$ are generated setting probability equal to 0.8 for a bit being 1. The last row population individual $p_{n_{p}}$ is set to $p_{all}$ and is defined as a population individual representing an ’All-feature’ set in the optimization space. This tunes the DE optimization process to find a final subset of optimized and reduced features that achieves even better fitness than the all feature set and is mathematically represented in Equation (2).   

(2) $$p_{n_{p}}=p_{all}={\left[\begin{array}{ccccccc} 1 & 1 & 1 & . & . & . & 1 \end{array}\right]}_{1\;x\;n_{f}}$$

The number of individuals $n_{p}$ is chosen as 50 so that it is large enough to avoid stagnancy and small enough to avoid excessive computing time [47,48].

##### Fitness Evaluation

The fitness function, *fit* in this case, is modeled as the k-category MCC [36,49] mathematically expressed as Equation (3) considering one versus rest strategy taking all 8 classes one by one as positive (P) and the rest of 7 classes as negative class (N). All feature subsets represented by **p** in **P** are selected from the dataset and individually trained using PNN as explained in Section 2.2.4 and *fit* is calculated on the testing subset.

(3) $${\mathrm{MCC}}_{k}=\frac{TP\cdot TN+FP\cdot FN}{\sqrt{\left(TP+FP)(TP+FN)(TN+FP)(TN+FN\right)}}$$

Here, *TP* = number of samples for which positive class was correctly identified, *TN* = number of samples for which negative class was correctly identified, *FP* = number of samples for which positive class was wrongly identified and *FN* = number of samples for which negative class was wrongly identified and *k* denotes the number of classes and k=8 for the current problem. Hence, *FP* and *FN* represent misclassifications or error made by the classification algorithm. Mean calculated over MCC individually for 8 classes is modeled as *fit*. A maximization of *fit* is carried out to find the optimum combination of features. Maximization of the defined fitness function is carried out using maximum 200 generations.

(4) $$fit=max(mean\left(MCC_{k}\right))$$

##### Crossover

Randomly selecting two different individuals $p_{i1}$ and $p_{i2}$ from P, a 1-point crossover is performed where, i1, i2 are randomly generated index values between 1 and $n_{f}$ with crossover probability (CR=0.8). The population individual $v_{i}$ obtained after the crossover operation is called an offspring. Similarly, an offspring vector is created corresponding to every row in P to create a trial population matrix V.

##### Mutation

A bit-flip is performed with mutation probability (MR=0.2) for all $v_{i}$’s in V. Hence, currently there exists a parent population $P^{G}$ and an offspring population $V^{G+1}$ (after crossover and mutation) both of size $n_{p}$ x $n_{f}$.

##### Selection

The fitness function *fit* for each individual in the V is calculated using Equation (4). Applying the current-to-best strategy, if $v_{i}$ shows a higher *fit* value than the corresponding $p_{i}$, then $p_{i}$ in the P is replaced with $v_{i}$. Otherwise, the $p_{i}$ retains its position. This comparison and replacement process is repeated for every ($p_{i}$, $v_{i}$) pair an evolved version of P is obtained at the end of the generation. This process evolves and accumulates better individuals until the maximum number of generations, i.e., 200 is reached. After looping through all generations every individual in the P is replaced with the best possible candidate, i.e., having the highest *fit* value. $p_{sel}$ with the best *fit* in the end P is selected as the optimum feature subset with 1’s representing the selected features out of total $n_{f}$.

##### Termination

The process terminates if the maximum number of given generations 200 is reached or *fit* becomes stagnant for a consecutive 20 generations. For every new generation, a new V is generated using the updated P. Hence, crossover and mutation occur in every generation. The default control parameters are summarized in Table 1.

#### 2.2.4. Disease-Based Classification

Training and testing subsets composed of optimized subset of features $p_{sel}$ obtained in the last step are now extracted from complete training and testing subsets and can now be used to classify unseen beats using PNN [50]. The PNN consists of an input layer, a pattern layer, a summation layer, and a output layer. This architecture is illustrated in Figure 4 (Step 4). The neurons of the input layer convey the input features $a={\left[a_{1},a_{2},\ldots a_{j},\ldots ,a_{n_{s}}\right]}^{T}$ to the neurons of the pattern layer directly, where $n_{s}$ represents the number of optimized features in $p_{sel}$ and $n_{s}<=n_{f}$.

In the pattern layer of PNN, Gaussian function is used to calculate the output of the neuron $a_{ko}$ as in Equation (5) using the input vector **a** transferred down from the input layer:

(5) $$g_{ki}\left(a\right)=\frac{1}{\sqrt{{\left(2\pi \sigma^{2}\right)}^{n_{f}}}}\exp\left(-\frac{{∥a-a_{\mathrm{ki}}∥}^{2}}{2\sigma^{2}}\right)$$

where, $a_{ki}$ is the vector of neurons, σ defines the standard deviation also called spread for the Gaussian function and $n_{s}$ is the size/dimension of the pattern vector **a**. $∥a-a_{\mathrm{ki}}∥$ is the Euclidean distance between *a* and $a_{ki}$. The neurons in the summation layer calculate the maximum likelihood of the pattern vector **a** being categorized into class *k* by averaging the output of all neurons in the pattern layer that belong to the same class as mentioned in Equation (6).

(6) $$s_{k}\left(a\right)=\frac{1}{(\sqrt{{\left(2\pi \sigma^{2}\right)}^{n_{b}}}}\frac{1}{n_{k}}\sum_{1}^{n_{k}}\exp\left(-\frac{{∥a-a_{\mathrm{ki}}∥}^{2}}{2\sigma^{2}}\right)$$

where $n_{k}$ is the total number of the samples in class *k*. The neuron in the decision layer applies Bayes’s decision rule to determine the class belongingness of the pattern *a* by Equation (7).

(7) $$c\left(a\right)=max(p_{k}\left(a\right))$$

where *k* denotes the number of classes in the training samples and c(a) is the estimated class of the pattern **a**. In this paper, the output of the PNN is represented as the *Lb* of the eight types of ECG beats (i.e., NORM, LBBB, RBBB, PVC, APC, VESC, VFLT and PACE are labeled as ‘1’, ‘2’, ‘3’, ‘4’, ‘5’, ‘6’, ‘7’, and ‘8’, respectively). The detailed pseudocode for the proposed DE-based feature optimization and PNN classification strategy is given in Algorithm 1.
**Algorithm 1** Pseudocode of DE-PNN algorithm for feature optimization in heartbeat classification problem**Input:** dataset constructed using beat samples (DS) and associated class labels (Lb) as in
Section 2.1population size $\left(n_{p}\right)$ = 50maximum number of generations (maxGen) = 200crossover probability (CR) = 0.8mutation rate (MR) = 0.2feature size $\left(n_{f}\right)$ = 253current generation (gen) = 1start DE: DS, Lb, $n_{p}$, maxGen, CR, MR// Population Initialization %for *i* = 1 $n_{p}$for *j* = 1 $n_{f}$if rand[0,1] > 0.5$P_{i,j}^{G=0}$ = 1endifendforendfor// Replace the last population individual with an all feature vector $P_{n_{p},j}^{G=0}$ = 1 1 1 …1 ${}_{1\;x\;n_{f}}$// For each population individual (i.e., bit string $P_{i}$) calculate ‘fit’representing classification performance metric of PNN as in Equation (2)for i = 1 $n_{p}$${fit|}_{DS(P_{i})}^{G+1}$endfor // Generate test vectorswhile gen <= maxGenfor j = 1 $n_{f}$// Select two separate random population individuals (i.e., bit strings $P_{i1}\;and\;P_{i2}$)for i = 1 $n_{p}$if rand(0,1) < CRi1 = rand[1,$n_{p}$], i2 = rand[1,$n_{p}$] and ≠ i1ri = rand[1,$n_{f}$]$V_{i,j}^{G+1}=\left[P_{i1,j}^{G}\left[1:ri\right],\;\;P_{i2,j}^{G}\left[ri+1:n_{f}\right]\right]$endifendforendfor// Select the individual with better ’fit’if ${fit|}_{DS(V_{i})}^{G+1}{>fit|}_{DS(P_{i})}^{G+1}$$P_{i,j}^{G}=V_{i,j}^{G+1}$else$P_{i,j}^{G}=P_{i,j}^{G}$endifgen = gen + 1endwhileendprocedure// Return optimized combination of features
**Output:**$p_{sel}=p_{best}$ with maximum ’fit’ value as in Equation (3)

## 3. Performance Evaluation

Out of the 108,700 beat samples, 50% were selected as the training subset and the remaining 50% as the testing subset. Table 2 summarizes the details of the available beat samples from each class. All the available class samples in the MIT-BIH database are used in the current test to keep the arrhythmia instance ratio as close to real as possible.

Classification metrics; Matthew’s correlation coefficient (*MCC*), macro F1-score (*Macro-F1*) and accuracy (*Acc*) and area under the curve (*AUC*) have been reported. *MCC*, *Macro-F1*, *Acc* and additionally, sensitivity (*Sen*), and specificity (*Spe*) are reported according to Equation (3), Equations (8)–(11) with *fit* modeled as *MCC*.

All the definitions mentioned below follow a one-versus-rest strategy [51]. Each classification measure is calculated for each of the eight classes (taking one class as positive and all the rest as negative) and then averaged to represent the mean classification measure. The PNN classification was performed for *All features* set (as the exact solution) and *Optimized features* subset obtained after DE. Hence, all measures are reported for both *All features* and *Optimized features* cases to present a comparison between classification improvement and feature reduction achieved using the proposed method. Here, *TP*, *TN*, *FP*, and *FN* follow the same definition as mentioned in ’Fitness Evaluation’ part.

(8) $$\mathrm{Acc}=\frac{TP+TN}{TP+TN+FP+FN}$$

(9) $$\mathrm{Sen}=\frac{TP}{TP+FN}$$

(10) $$\mathrm{Spe}=\frac{TN}{TN+FP}$$

(11) $$F1=\frac{2\cdot TP}{2\cdot TP+FP+FN}$$

(12) $$Macro-F1=\frac{1}{N}\sum_{c=1}^{N}F1_{c},$$

## 4. Results

**Figure 6:**
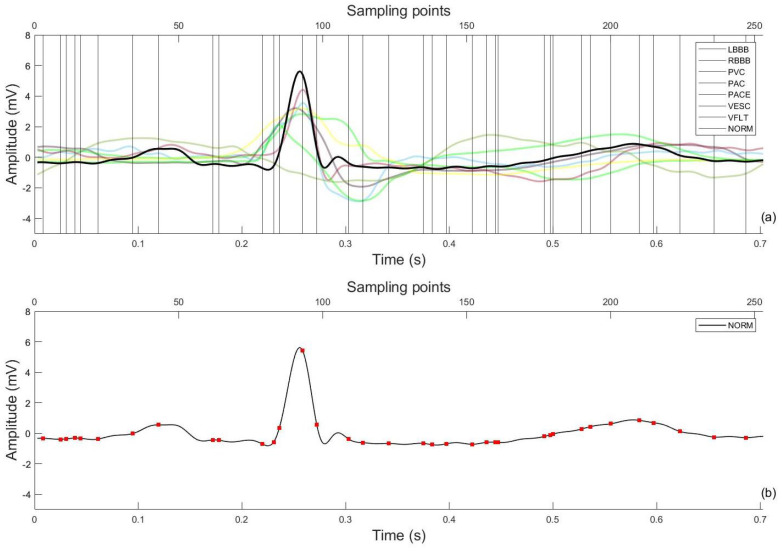
Selected feature scan after DE with all beat classes (**a**), feature points on 1 representative beat (**b**).

Table 3 shows a comparison of the proposed DE-PNN algorithm with the selected `All feature’ standard. The confusion matrices for both are reported in Table 4. The optimized features which result in the maximum *MCC* are plotted in Figure 6. The average number of generations by which the optimization is achieved was 78 ± 12 (10 trials). After an average 78 generations, the fitness value becomes stagnant meaning the fitness function has achieved its maximum value and is no longer improving.

Using the DE-PNN scheme, the best and worst were the accuracy of 99.84% for VESC and 95.41% for NORM, respectively. The DE-PNN scheme could classify NORM with an accuracy of 99.45%, PVC with 99.18%, PACE with 100.00%, RBBB with 99.94%, LBBB with 99.80%, APC with 99.76%, VFLT with 99.61%, and VESC with 99.94%. These results demonstrate the abilities of the above-mentioned ECG arrhythmia classification schemes to classify the eight ECG beats effectively. The overall accuracy of the DE-PNN scheme, the DE-PNN scheme, and the DE-PNN scheme were 99.61%, 98.26%, and 99.71%, respectively, as reported in Table 5.

**Figure 7:**
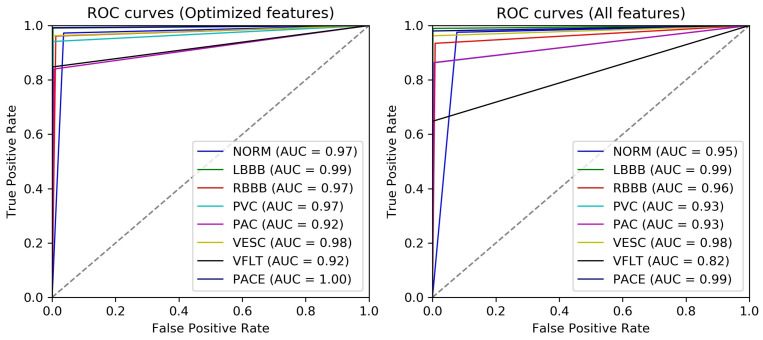
ROC curves of 8 classes for Optimized feature subset (left panel) and All feature set (right panel).

To analyze the efficacy of optimized features in distinguishing between simulated cardiac conditions, the receiver operating characteristic (ROC) is plotted using a one-versus-all class strategy and area under the curve (AUC) is calculated. By analogy, the higher the AUC, the better the capability of recognition of the particular class by the classification algorithm. Figure 7 shows the ROCs and AUCs of every class in the case of optimized and all features. The AUC for all arrhythmia classes except paced beat has increased with maximum AUC improvement for VFLT (10%) which is the rarest class in the currently used dataset and secondarily PVC (4%), both representing critical pathological conditions. Overall, the recognition for all classes has improved or stayed consistent with 85.77% reduction in number of features.

## 5. Discussion

The proposed method presents an accurate and computationally efficient arrhythmia classification method using direct ECG amplitude signal features. More than 100,000 ECG heartbeats are obtained with eight types of ECG beats including one normal and seven arrhythmic beat types. Feature optimization is performed by modeling optimization input as binary vectors representing different feature combinations using DE. An optimized feature subset is obtained which is then used with a simple PNN classifier. The proposed method achieved 85.77% reduction in directly acquired features with comparable classification performance. Figure 6 shows the optimized and selected 36 out of 253 (total amplitude feature points). The higher classification performance achieved could be due to better beat definition (250 ms before and 450 ms after the R-peak positions) as compared to [52] which arbitrarily used 200 samples around the R-peak. Our definition makes sure the inclusion of important physiological characteristics necessary to distinguish between the currently classified arrhythmia types which are most ventricular types. Furthermore, on the algorithm design level, adding an all feature combination to the solution space pushes the optimization process to find a solution better than the *All features* scenario.

Moreover, we compared the classification performance of the proposed DE-PNN scheme for ECG arrhythmia classification with those of other schemes simultaneously utilizing different feature reduction methods and neural classifiers presented in the literature as summarized in Table 6. Jun et al. [53] used the same direct ECG amplitude features as used in this work and presented a comparison between 2D-CNN, AlexNet, and VGGNet models. All three of the models were deployed using TensorFlow [54] which is a deep learning Python library proposed by Google especially for GPGPUs and yet used two Intel Xeon E5 CPUs and two NVIDIA K20m GPUs to reduce the learning time. All tested classifiers had complex architectures implying extremely high computational cost with no feature optimization/reduction function which is not suitable for continuous monitoring using wearable sensing modality. Yildrim et al. [19], Tuncer et al. [20], and Elhaj et al. [10] used wavelet features with multiple different combination of features to perform arrhythmia classification adding feature computation layer in the processing algorithms performing optimizations focused on classifier parameters rather than feature engineering.

DE-PNN aimed at searching for the optimum feature combination that provides maximum recognition capability for arrhythmic heartbeats removing redundant and selecting highly discriminating features. Overall, the achieved ECG arrhythmia classification result indicates that the detection of arrhythmia using 14.23% (85.77% reduced) features of a complete ECG heartbeat can be an effective approach to help general physicians and cardiology specialists to diagnose critical cardiovascular diseases in continuous and long-term, online or offline monitoring scenarios particularly well-suited for a wearable sensing setting. For future work, the current algorithm may be extended to recognize 16 classes (1 normal and 15 arrhythmic) for which the annotations are available with the MIT-BIH dataset. A future DE optimization might focus on a multi-objective approach to maximize arrhythmia recognition whilst minimizing percentage signal distortion (accuracy and compression being the two objective functions) to make the ECG signal reproducible for clinical analysis.

## Figures and Tables

**Table 1:** DE control parameters summary.

Parameter	Value
Population size	50
Population type	Binary bits
Crossover	1-point crossover
Mutation	Uniform
Selection scheme	Current-to-best
Population individual length	253
Maximum number of generations	200
Crossover probability	0.8
Mutation probability	0.2

**Table 2:** MIT-BIH data selection details.

Beat Class	Training	Testing	Total
NORM	36,907	36,907	73,814
LBBB	4031	4031	8062
RBBB	4533	4533	9066
PVC	3363	3363	6726
PAC	1270	1271	2541
VESC	53	53	106
VFLT	236	236	472
PACE	3506	3507	7013
Total	53,899	53,901	107,800

**Table 3:** Classification test result.

Features	NumFeat	MCC	Acc	Macro-F1	AUC
All	253	0.1248	99.05	92.44	0.8242
Optimized	36	0.1250	99.33	94.56	0.8370
Difference	−217	+0.0002	+0.28	+2.12	+0.0128

**Table 4:** Confusion matrices for testing subset with Optimized and All features with *fit* = MCC for 1 normal and 7 arrhythmia classes.

Optimized Features								
**T/P**	**NORM**	**LBBB**	**RBBB**	**PVC**	**PAC**	**VESC**	**VFLT**	**PACE**
NORM	7467	4	76	21	40	1	1	0
LBBB	7	778	0	11	2	2	0	0
RBBB	80	0	1141	2	13	1	3	0
PVC	50	11	5	2409	10	5	11	0
PAC	105	0	15	4	657	0	0	0
VESC	2	0	0	0	0	51	0	0
VFLT	15	1	11	22	1	3	147	0
PACE	0	0	0	0	0	0	0	800
**All Features**								
**T/P**	**NORM**	**LBBB**	**RBBB**	**PVC**	**PAC**	**VESC**	**VFLT**	**PACE**
NORM	7481	3	71	6	49	0	0	0
LBBB	7	785	0	4	2	2	0	0
RBBB	87	0	1146	2	2	1	2	0
PVC	109	9	4	2364	5	6	3	1
PAC	79	0	10	1	691	0	0	0
VESC	2	0	0	0	0	51	0	0
VFLT	23	1	12	19	3	4	137	1
PACE	1	0	0	0	0	0	0	799

**Table 5:** Classification results for testing subset with Optimized and All features with *fit* = MCC for 1 normal and 7 arrhythmia classes.

Optimized Features				
**Class**	**Acc (%)**	**Sen (%)**	**Spe (%)**	**F1 (%)**
NORM	96.73	97.24	96.37	96.10
LBBB	99.65	99.06	99.73	98.60
RBBB	98.50	96.13	98.84	94.12
PVC	98.93	94.13	99.62	95.66
PAC	98.12	84.00	99.40	88.14
VESC	99.90	96.22	99.93	94.44
VFLT	99.28	84.78	99.86	90.06
PACE	99.90	99.20	99.96	99.39
Average	99.33	93.84	99.21	94.56
**All Features**				
**Class**	**Acc (%)**	**Sen (%)**	**Spe (%)**	**F1 (%)**
NORM	94.46	97.52	92.30	93.58
LBBB	99.73	98.93	99.84	98.93
RBBB	98.44	93.46	99.14	93.71
PVC	97.97	86.26	99.64	91.38
PAC	98.34	86.40	99.42	89.62
VESC	99.91	96.22	99.95	95.32
VFLT	98.62	64.78	99.96	78.21
PACE	99.80	98.00	99.96	98.79
Average	99.05	90.20	98.77	92.44

**Table 6:** Comparison of the proposed DE-PNN scheme with latest literature.

Research	Feature Type	#Classes	Feature Selection	Classification	Accuracy (%)
DE-PNN	Morphology	8	DE	PNN	99.33
[53]	Morphology	8	None	CNN	98.90
[53]	Morphology	8	None	AlexNet	98.80
[53]	Morphology	8	None	VGGNet	98.70
[19]	Morphology	5	convolutional AE	LSTM	99.00
[29]	Wavelet	5	PSO	LS-SVM, RF	98.95
[10]	HOS+Wavelet	5	ICA+PCA	SVM+NN	98.91
[28]	PSD+DFT	17	GA	SVM, kNN, PNN, and RBFNN	98.85
[55]	DCT+weighted inter-beat	5, 15	none	SVM	98.46
[20]	Multilevel wavelet	17	NCA	1-NN	95.00
[12]	k-medoids vector quantization	4	none	parallel regression NN	95.00
[16]	Morphology	5	none	9-layer Deep CNN	94.03
[56]	Temporal vectorcardiogram	3	PSO	SVM	92.40

## Data Availability

The data used in this study belongs to MIT-BIH Arrhythmia database and is available opensource at Physionet (https://physionet.org/content/mitdb/1.0.0/ accessed on 15 December 2020).

## References

[1] Baig M.M., Gholamhosseini H., Connolly M.J. (2013). A comprehensive survey of wearable and wireless ECG monitoring systems for older adults. Med. Biol. Eng. Comput..

[2] Davenport C., Cheng E.Y.L., Kwok Y.T.T., Lai A.H.O., Wakabayashi T., Hyde C., Connock M. (2006). Assessing the diagnostic test accuracy of natriuretic peptides and ECG in the diagnosis of left ventricular systolic dysfunction: A systematic review and meta-analysis. Br. J. Gen. Pract..

[3] Pollonini L., Rajan N.O., Xu S., Madala S., Dacso C.C. (2012). A novel handheld device for use in remote patient monitoring of heart failure patients—Design and preliminary validation on healthy subjects. J. Med. Syst..

[4] López G., Custodio V., Moreno J.I. (2010). LOBIN: E-textile and wireless-sensor-network-based platform for healthcare monitoring in future hospital environments. IEEE Trans. Inf. Technol. Biomed..

[5] Yoo J., Yan L., Lee S., Kim H., Yoo H.J. (2009). A wearable ECG acquisition system with compact planar-fashionable circuit board-based shirt. IEEE Trans. Inf. Technol. Biomed..

[6] Sahoo S., Dash M., Behera S., Sabut S. (2020). Machine learning approach to detect cardiac arrhythmias in ECG signals: A survey. IRBM.

[7] Sree V., Mapes J., Dua S., Lih O.S., Koh J.E., Ciaccio E.J., Acharya U.R. (2021). A novel machine learning framework for automated detection of arrhythmias in ECG segments. J. Ambient. Intell. Humaniz. Comput..

[8] Fujita H., Cimr D. (2019). Decision support system for arrhythmia prediction using convolutional neural network structure without preprocessing. Appl. Intell..

[9] Sayantan G., Kien P., Kadambari K. (2018). Classification of ECG beats using deep belief network and active learning. Med. Biol. Eng. Comput..

[10] Elhaj F.A., Salim N., Harris A.R., Swee T.T., Ahmed T. (2016). Arrhythmia recognition and classification using combined linear and nonlinear features of ECG signals. Comput. Methods Programs Biomed..

[11] Afkhami R.G., Azarnia G., Tinati M.A. (2016). Cardiac arrhythmia classification using statistical and mixture modeling features of ECG signals. Pattern Recognit. Lett..

[12] Liu T., Si Y., Wen D., Zang M., Lang L. (2016). Dictionary learning for VQ feature extraction in ECG beats classification. Expert Syst. Appl..

[13] Shen C.P., Kao W.C., Yang Y.Y., Hsu M.C., Wu Y.T., Lai F. (2012). Detection of cardiac arrhythmia in electrocardiograms using adaptive feature extraction and modified support vector machines. Expert Syst. Appl..

[14] Qin Q., Li J., Zhang L., Yue Y., Liu C. (2017). Combining low-dimensional wavelet features and support vector machine for arrhythmia beat classification. Sci. Rep..

[15] Zhai X., Tin C. (2018). Automated ECG classification using dual heartbeat coupling based on convolutional neural network. IEEE Access.

[16] Acharya U.R., Oh S.L., Hagiwara Y., Tan J.H., Adam M., Gertych A., San Tan R. (2017). A deep convolutional neural network model to classify heartbeats. Comput. Biol. Med..

[17] Oh S.L., Ng E.Y., San Tan R., Acharya U.R. (2018). Automated diagnosis of arrhythmia using combination of CNN and LSTM techniques with variable length heart beats. Comput. Biol. Med..

[18] Zhang Y., Zhang Y., Lo B., Xu W. (2019). Wearable ECG signal processing for automated cardiac arrhythmia classification using CFASE-based feature selection. Expert Syst..

[19] Yildirim O., Baloglu U.B., Tan R.S., Ciaccio E.J., Acharya U.R. (2019). A new approach for arrhythmia classification using deep coded features and LSTM networks. Comput. Methods Programs Biomed..

[20] Tuncer T., Dogan S., Pławiak P., Acharya U.R. (2019). Automated arrhythmia detection using novel hexadecimal local pattern and multilevel wavelet transform with ECG signals. Knowl.-Based Syst..

[21] Wang J.S., Chiang W.C., Hsu Y.L., Yang Y.T.C. (2013). ECG arrhythmia classification using a probabilistic neural network with a feature reduction method. Neurocomputing.

[22] Alonso-Atienza F., Morgado E., Fernandez-Martinez L., García-Alberola A., Rojo-Alvarez J.L. (2013). Detection of life-threatening arrhythmias using feature selection and support vector machines. IEEE Trans. Biomed. Eng..

[23] Chen Y.H., Yu S.N. (2012). Selection of effective features for ECG beat recognition based on nonlinear correlations. Artif. Intell. Med..

[24] Asl B.M., Setarehdan S.K., Mohebbi M. (2008). Support vector machine-based arrhythmia classification using reduced features of heart rate variability signal. Artif. Intell. Med..

[25] Haseena H.H., Mathew A.T., Paul J.K. (2011). Fuzzy clustered probabilistic and multi layered feed forward neural networks for electrocardiogram arrhythmia classification. J. Med. Syst..

[26] Ceylan R., Özbay Y. (2007). Comparison of FCM, PCA and WT techniques for classification ECG arrhythmias using artificial neural network. Expert Syst. Appl..

[27] Polat K., Güneş S. (2007). Detection of ECG Arrhythmia using a differential expert system approach based on principal component analysis and least square support vector machine. Appl. Math. Comput..

[28] Pławiak P. (2018). Novel methodology of cardiac health recognition based on ECG signals and evolutionary-neural system. Expert Syst. Appl..

[29] Yildirim Ö., Baloglu U.B. (2018). Heartbeat type classification with optimized feature vectors. Int. J. Optim. Control. Theor. Appl. (IJOCTA).

[30] Houssein E.H., Ewees A.A., ElAziz M.A. (2018). Improving twin support vector machine based on hybrid swarm optimizer for heartbeat classification. Pattern Recognit. Image Anal..

[31] Li H., Yuan D., Ma X., Cui D., Cao L. (2017). Genetic algorithm for the optimization of features and neural networks in ECG signals classification. Sci. Rep..

[32] Daskalaki S., Kopanas I., Avouris N. (2006). Evaluation of classifiers for an uneven class distribution problem. Appl. Artif. Intell..

[33] Sun K.W., Lee C.H. (2017). Addressing class-imbalance in multi-label learning via two-stage multi-label hypernetwork. Neurocomputing.

[34] Maalouf M., Siddiqi M. (2014). Weighted logistic regression for large-scale imbalanced and rare events data. Knowl.-Based Syst..

[35] Yu H., Sun C., Yang X., Yang W., Shen J., Qi Y. (2016). ODOC-ELM: Optimal decision outputs compensation-based extreme learning machine for classifying imbalanced data. Knowl.-Based Syst..

[36] Matthews B.W. (1975). Comparison of the predicted and observed secondary structure of T4 phage lysozyme. Biochim. Biophys. Acta (BBA)-Protein Struct..

[37] Shi L., Campbell G., Jones W.D., Campagne F., Wen Z., Walker S.J., Su Z., Chu T.M., Goodsaid F.M., Pusztai L. (2010). The MicroArray Quality Control (MAQC)-II study of common practices for the development and validation of microarray-based predictive models. Nat. Biotechnol..

[38] Storn R., Price K. (1997). Differential evolution—A simple and efficient heuristic for global optimization over continuous spaces. J. Glob. Optim..

[39] Zaman M.A., Chowdhury S. (2013). Modified Bézier curves with shape-preserving characteristics using Differential Evolution optimization algorithm. Adv. Numer. Anal..

[40] Liu X.F., Zhan Z.H., Zhang J. (2021). Resource-aware distributed differential evolution for training expensive neural-network-based controller in power electronic circuit. IEEE Trans. Neural Netw. Learn. Syst..

[41] Saporetti C.M., Goliatt L., Pereira E. (2021). Neural network boosted with differential evolution for lithology identification based on well logs information. Earth Sci. Inform..

[42] Sikder U., Zaman M.A. (2016). Optimization of multilayer antireflection coating for photovoltaic applications. Opt. Laser Technol..

[43] Moody G.B., Mark R.G. (2001). The impact of the MIT-BIH arrhythmia database. IEEE Eng. Med. Biol. Mag..

[44] Goldberger A.L., Amaral L.A., Glass L., Hausdorff J.M., Ivanov P.C., Mark R.G., Mietus J.E., Moody G.B., Peng C.K., Stanley H.E. (2000). PhysioBank, PhysioToolkit, and PhysioNet: Components of a new research resource for complex physiologic signals. Circulation.

[45] Zhu H., Pan Y., Wu F., Huan R. (2019). Optimized Electrode Locations for Wearable Single-Lead ECG Monitoring Devices: A Case Study Using WFEES Modules Based on the LANS Method. Sensors.

[46] Marinucci D., Sbrollini A., Marcantoni I., Morettini M., Swenne C.A., Burattini L. (2020). Artificial Neural Network for Atrial Fibrillation Identification in Portable Devices. Sensors.

[47] Neri F., Tirronen V. (2010). Recent advances in differential evolution: A survey and experimental analysis. Artif. Intell. Rev..

[48] Yang M., Cai Z., Li C., Guan J. An improved adaptive differential evolution algorithm with population adaptation. Proceedings of the 15th Annual Conference on Genetic and Evolutionary Computation.

[49] Gorodkin J. (2004). Comparing two K-category assignments by a K-category correlation coefficient. Comput. Biol. Chem..

[50] Specht D.F. (1990). Probabilistic neural networks. Neural Netw..

[51] Xu J. (2011). An extended one-versus-rest support vector machine for multi-label classification. Neurocomputing.

[52] Wang T., Lu C., Sun Y., Yang M., Liu C., Ou C. (2021). Automatic ECG classification using continuous wavelet transform and convolutional neural network. Entropy.

[53] Jun T.J., Nguyen H.M., Kang D., Kim D., Kim D., Kim Y.H. (2018). ECG arrhythmia classification using a 2-D convolutional neural network. arXiv.

[54] Abadi M., Agarwal A., Barham P., Brevdo E., Chen Z., Citro C., Corrado G.S., Davis A., Dean J., Devin M. (2015). TensorFlow: Large-Scale Machine Learning on Heterogeneous Systems. https://arxiv.org/abs/1603.04467.

[55] Chen S., Hua W., Li Z., Li J., Gao X. (2017). Heartbeat classification using projected and dynamic features of ECG signal. Biomed. Signal Process. Control..

[56] Garcia G., Moreira G., Menotti D., Luz E. (2017). Inter-patient ECG heartbeat classification with temporal VCG optimized by PSO. Sci. Rep..

